# Positive mental health and mindfulness as protective factors against addictive social media use during the COVID-19 outbreak

**DOI:** 10.1371/journal.pone.0277631

**Published:** 2022-11-30

**Authors:** Julia Brailovskaia, Jürgen Margraf

**Affiliations:** Department of Clinical Psychology and Psychotherapy, Mental Health Research and Treatment Center, Ruhr-Universität Bochum, Bochum, Germany; National Cheng Kung University College of Medicine, TAIWAN

## Abstract

The outbreak of COVID-19 caused high psychological burden for many people. Some people tend to excessive social media use (SMU) to escape the negative emotions which can foster addictive tendencies. The present study investigated positive mental health (PMH) and mindfulness as protective factors that could reduce the risk for addictive SMU. Data of 1,049 participants from Germany were assessed via online surveys in autumn 2021. The current results reveal a positive relationship between COVID-19 burden and addictive SMU. Both were negatively linked to PMH and mindfulness. In a moderated mediation analysis, the relationship between COVID-19 burden and addictive SMU was mediated by PMH. Mindfulness moderated the association between PMH and addictive SMU. The COVID-19 situation can be burdensome and contribute to dysfunctional coping strategies such as addictive SMU. However, PMH and mindfulness serve as protective factors. The protective effect of mindfulness could be especially important for persons with low PMH.

## Introduction

The outbreak of the coronavirus disease 2019 (COVID-19) [1] resulted in significant changes of people’s everyday routine. Curfews, lockdowns, restrictions of face-to-face contacts (“social distancing”), worries about the own health and the health of close others were experienced as a high psychological burden by many people [2]. To escape the negative emotions, some persons tended to dysfunctional coping strategies such as excessive social media use (SMU) [3]. On Instagram, Twitter, and Facebook, they could engage in various social interactions that were restricted offline. In the short-term, excessive SMU could reduce the feelings of burden caused by the COVID-19 situation and foster positive emotions [4]. However, it the longer-term, the positive experiences could contribute to the development of an emotional bond to social media that is closely associated with an obsessive need to stay permanently online [5].

This phenomenon has been termed as addictive SMU. Six typical characteristics describe addictive SMU: salience, tolerance, mood modification, relapse, withdrawal, conflicts [6]. Addictive SMU does not belong to recognized formal psychiatric disorders (e.g., International Classification of Diseases, ICD-11 [7]). And some researchers emphasized that intensive online activity should not be over pathologized [8]. However, recent findings showed that addictive SMU is closely linked to a decrease of life satisfaction and to an increase of interpersonal problems, depression symptoms, and suicide-related outcomes [9,10]. Reduction of time spent daily on SMU reduced the addictive tendencies as well as symptoms of depression and anxiety [11–13]. Recent research emphasized the relevance of the addictive SMU issue especially in the context of the global COVID-19 spread [14]. Several studies reported an increase of addictive SMU since the pandemic outbreak [15,16]. In particular people, who had to stay in home quarantine due to the COVID-19 infection, had enhanced levels of the addictive tendencies that were accompanied by increased levels of depression and anxiety symptoms [17]. Furthermore, research that focused on schoolchildren described a close positive association between addictive SMU and psychological distress during the course of the pandemic [18,19]. In addition, the pre-pandemic level of addictive SMU predicted the level of psychological distress up to several months after the COVID-19 outbreak positively in schoolchildren [20,21]. The findings underly the potential negative effect of addictive SMU on mental health.

Against these findings and considering the uncertainty about the future course of the pandemic and its longer-term consequences for one’s everyday life, it is important to identify protective factors that could reduce the risk for addictive SMU in the context of COVID-19. Of note, most previous studies focused only on factors that could foster the addictive tendencies in the context of COVID-19 [5,22,23]. Therefore, only little is known about potential protective factors, so far.

Following available research [24], positive mental health (PMH)–that is emotional, social, and psychological well-being [25]–might be one of the protective factors. PMH is a well-known protective factor against psychological distress, anxiety symptoms, and suicide-related outcomes [26–28]. Moreover, it was negatively associated with addictive SMU [29]. PMH can confer resilience and strengthens functional coping strategies in uncertain situations [28]. Thus, we could hypothesize that PMH might also reduce the risk of addictive SMU that results from COVID-19 burden. However, recent research described a decrease of PMH since the pandemic outbreak [30]. Therefore, it seems reasonable to consider an additional protective factor against addictive SMU.

Király, Potenza (14) presented a consensus guidance that should contribute to a reduction of problematic/addictive online activity during the pandemic. In addition to strategies such as the regulation of time spent online and the maintenance of daily routine including a regular sleep and food intake, the authors emphasized mindfulness as a potential protective factor. Mindfulness has been defined as increased attention to and nonjudgment awareness of the current moment [31]. It is positively associated with self-awareness of own behavior and self-efficacy [32,33]. Furthermore, it fosters functional coping strategies and reduces the risk for maladaptive and self-destructive behavior in stressful situation [34]. Therefore, mindfulness training is often included in the treatment process of problematic alcohol and illicit substance use [35]. Several studies described a negative relationship between mindfulness and addictive SMU [36–39]. Two studies that assessed data during the pandemic emphasized that mindfulness could reduce the negative influence of excessive SMU on symptoms of stress and depression [3,4]. Notably, mindfulness training also promoted PMH and fostered its protective effect [40].

**Fig 1:**
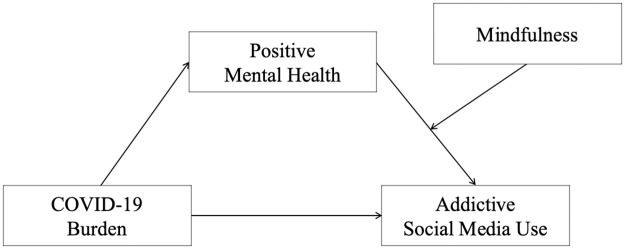
Moderated mediation model with COVID-19 burden (predictor), positive mental health (mediator), mindfulness (moderator), and addictive social media use (outcome).

Against the presented background, the aim of the present study was to investigate the relationship between COVID-19 burden, addictive SMU, PMH and mindfulness. And thus, to contribute to the explanation of potential factors and mechanisms that could reduce the risk for addictive SMU during the pandemic. We hypothesized a positive relationship between COVID-19 burden and addictive SMU (Hypothesis 1a). In contrast, we expected COVID-19 burden to be negatively related to PMH (Hypothesis 1b) and to mindfulness (Hypothesis 1c). Also, we assumed that addictive SMU is negatively linked to PMH (Hypothesis 2a) and mindfulness (Hypothesis 2b). And we assumed a positive relationship between PMH and mindfulness (Hypothesis 3). Furthermore, previous research described a buffering effect of PMH on the influence of stressful events on adjustment disorder symptoms [28]. Therefore, we hypothesized that PMH could mediate the positive association between COVID-19 burden and addictive SMU (Hypothesis 4a). Considering that mindfulness training contributed to the protective effect of PMH [40], we furthermore hypothesized that mindfulness could moderate the negative link between PMH and addictive SMU (Hypothesis 4b). Specifically, the higher the level of mindfulness, the stronger the protective effect of PMH on addictive SMU. Fig 1 illustrates the hypothesized associations as a moderated mediation model [41].

## Materials and methods

### Procedure and participants

In October 2021, we have sent a collective e-mail invitation including a link of the online survey to 1,200 current or former students at a large university in the Ruhr region of Germany. They all had agreed to be contacted for research investigations. Participation was voluntary and compensated by course credits for students. The only requirement for participation was the membership on any form of social media. Overall, 1,102 (91.8%) people started the survey and 1,049 (87.4%) participants (71.6% women; age: *M* = 24.60, *SD* = 6.76, range: 18–72) completed the survey. There were no missing data in the completed survey. The current sample included 78.1% students, 21.4% employees, and 0.6% unemployed persons. Considering the marital status, 48.8% were singles, 42.0% were in a romantic relationship, and 9.2% were married. All participants were instructed and gave informed consent to participate via an online form. The Ethics Committee of the Faculty of Psychology of the Ruhr-Universität Bochum approved the implementation of the present study (approval number: 20110512). The dataset used in the present study is available in S1 Dataset.

### Measures

#### Psychological Burden caused by COVID-19

We used the COVID-19 Burden Scale (original German version: [42]) to assess the psychological burden caused by the COVID-19 situation. The six items (e.g., “I feel socially isolated”) are rated on a 7-point Likert-type scale (1 = *I do not agree*, 7 = *I totally agree*). The higher the sum score, the higher the burden. Current scale reliability: Cronbach’s *α* = .822.

#### Positive Mental Health (PMH)

The unidimensional Positive Mental Health Scale (PMH-Scale; original German version: [25]) measured PMH. Its nine items are rated on a 4-point Likert-type scale (e.g., “I enjoy my life”; 0 = *do not agree*, 3 = *agree*). Higher sum scores indicate higher levels of PMH. Current scale reliability: *α* = .923.

#### Addictive SMU

The brief version of the Bergen Social Media Addiction Scale (BSMAS; original version: [6]; German language version: [43]) assessed addictive SMU. This instrument includes six items (e.g., “Felt an urge to use social media more and more?”) according to the six core characteristics of addictive SMU that are rated on a 5-point Likert-type scale (1 = *very rarely*, 5 = *very often*). The higher the sum score, the higher the level of addictive SMU. Current scale reliability: *α* = .853.

#### Mindfulness

We assessed mindfulness with the brief version of the Freiburg Mindfulness Inventory (FMI-8; original German version: [44]). The eight items (e.g., “I am open to the experience of the present moment”) are rated on a 4-point Likert-type scale (1 = *rarely*, 4 = *almost always*). Higher sum scores indicate higher mindfulness. Current scale reliability: *α* = .834.

### Statistical analyses

Statistical analyses were conducted using SPSS 28 [45] and the macro Process version 4.0 (www.processmacro.org/index.html) [41]. After descriptive analyses, we calculated zero-order bivariate correlations to assess the relationship between COVID-19 burden, addictive SMU, PMH, and mindfulness.

Next, we ran a moderated mediation analysis (Process: model 14). The model included a conditional indirect effect (see Fig 1) and examined the multiple effects simultaneously (integration of the hypothesized mediation and moderation effects) [46,47]. The bootstrapping procedure (10,000 samples) that provides percentile bootstrap confidence intervals (*CI* 95%) revealed the moderated mediation effect. COVID-19 burden served as the predictor, PMH as the mediator, mindfulness as the moderator, and addictive SMU as the outcome; considering the rather young and female composition of the present sample, age and gender were included as covariates. The link between COVID-19 burden and PMH was denoted by path *a*; path *b* denoted the relationship between PMH and addictive SMU; the association between COVID-19 burden and addictive SMU after the inclusion of PMH and mindfulness in the model was denoted by path *c’* (the direct effect).

## Results

COVID-19 burden (*M* = 20.75, *SD* = 7.70, range:6–42) was significantly negatively correlated with PMH (*M* = 17.09, *SD* = 6.07, range:0–27), *r* = -.410, *p* < .001, and with mindfulness (*M* = 20.67, *SD* = 4.80, range:8–32), *r* = -.347, *p* < .001. In contract, COVID-19 burden was significantly positively correlated with addictive SMU (*M* = 12.39, *SD* = 5.28, range:6–30), *r* = .333, *p* < .001. Addictive SMU was significantly negatively correlated with PMH, *r* = -.379, *p* < .001, and with mindfulness, *r* = -.321, *p* < .001. PMH was significantly positively correlated with mindfulness, *r* = .708, *p* < .001.

As shown in Table 1, the moderated mediation analysis revealed a significant overall model, *F*(6,1042) = 57.813, *p* < .001. The explained variance of the overall model was *R*^*2*^ = .250. The direct effect (path *c’*) of COVID-19 burden on addictive SMU was significant (*p* < .001) after controlling for PMH, mindfulness, and their interaction. The conditional indirect effect of COVID-19 burden on addictive SMU through PMH was significant in people with low (that is one SD below the mean in the analysis = -4.805), medium (that is the mean in the analysis = 0) and high (that is one SD above the mean in the analysis = 4.805) levels of mindfulness. But the higher the mindfulness, the weaker the effect (low > medium > high mindfulness; see Table 1). As indicated by the index of moderated mediation, the test of moderated mediation was significant, revealing a significant moderated mediation effect (see Table 1).

**Table 1:** Moderated mediation model (outcome: Addictive social media use).

	ß	SE	t	*p*	95% *CI*
Path *a*: COVID-19 Burden → PMH	-.322	.022	-14.463	< .001	[-.365, -.278]
Path *b*: PMH → Addictive SMU	-.190	.035	-5.490	< .001	[-.258, -.122]
Interaction: PMH*Mindfulness → Addictive SMU	.016	.004	3.763	< .001	[.008, .025]
Path *c’* (direct effect): COVID-19 Burden → Additive SMU	.126	.020	6.204	< .001	[.086, .166]
*Conditional Indirect Effects*: *COVID-19 Burden* → *Addictive SMU*					
COVID-19 Burden → PMH → Addictive SMU					
Mindfulness:					
Low (one *SD* below mean = -4.805)	.086	.017			[.054, .121]
Medium (mean = 0)	.061	.014			[.035, .089]
High (one *SD* above mean = 4.805)	.036	.017			[.003, .070]
*Index of Moderated Mediation*	-.005	.002			[-.009, -.001]

*N* = 1,049; covariates: Age and gender; PMH = Positive Mental Health; SMU = Social Media Use; ß = Standardized Beta; SE = Standard Error; *t* = *t*-test; *p* = significance; *CI* = Confidence Interval; explained variance of the overall model: *R*^*2*^ = .250.

## Discussion

Online interaction via social media is a promising alternative when face-to-face interaction is restricted [48]. It allows intensive exchange despite the social distancing during the COVID-19 situation [49]. However, people who experience the pandemic outbreak as a heavy psychological burden are prone to excessive SMU and the development of addictive tendencies [22]. The present study identified promising factors and mechanisms that could reduce the risk for this negative development.

In line with previous research [5], COVID-19 burden was positively related to addictive SMU (confirmation of Hypothesis 1a). Notably, an escape into the online world can reduce negative emotions associated with the burden at least temporarily. However, this positive experience does not eliminate the reason of the burden. In addition, it can contribute to the development of an emotional bond to the social media [50]. Consequently, the person engages each time in intensive SMU when she/he feels overwhelmed by the COVID-19 situation and searches for some relief and positive emotions. In the longer-term, this can foster addictive tendencies [51].

As expected, COVID-19 burden was negatively associated with PMH (confirmation of Hypothesis 1b) and mindfulness (confirmation of Hypothesis 1c). This finding corresponds to recent research. Individuals who had high levels of PMH prior to the pandemic outbreak were at lower risk to experience COVID-19 burden than persons who had lower PMH levels [42]. In a similar way, persons with a high level of mindfulness were at lower risk for psychological distress, depression and fear caused by the COVID-19 situation [3,4].

Furthermore, PMH (confirmation of Hypothesis 2a) and mindfulness (confirmation of Hypothesis 2b) were negatively linked to addictive SMU. Individuals with high levels of PMH are self-confident, optimistic and tend to functional coping strategies in stressful situations [28]. Also mindfulness can decrease the negative influence of stressful experiences and fosters functional coping strategies in uncertain situations [33]. Thus, both PMH and mindfulness could reduce the tendency to escape into the online world as a dysfunctional coping strategy during the pandemic. And this could reduce a person’s risk for the development of addictive tendencies. Moreover, we found a positive association between PMH and mindfulness (confirmation of Hypothesis 3) which is in line with available literature [40]. Against this background, one could speculate that PMH and mindfulness could strengthen the protective influence of each other on addictive SMU.

The results of the moderated mediation model provide first insight into the mechanisms that could underly the relationship between COVID-19 burden, addictive SMU, PMH and mindfulness. PMH mediated the effect of COVID-19 burden on addictive SMU (confirmation of Hypothesis 4a). Moreover, mindfulness moderated the link between PMH and addictive SMU (partly confirmation of Hypothesis 4b). However, the direction of the moderation effect was unexpected. Mindfulness did not enhance the protective effect of PMH on addictive SMU (contradiction of Hypothesis 4b). Rather higher levels of mindfulness decreased the closeness of the relationship between PMH and addictive SMU. This result could be interpreted as follows: PMH is an important protective factor that can buffer potential negative effects of the pandemic such as addictive SMU [28]. Yet, a comparison of the PMH level between 2020 and 2021 revealed a significant decrease due to the burdensome experiences since the pandemic outbreak [30]. The decrease in PMH could increase the risk for addictive SMU. However, mindfulness could reduce this negative effect and specifically protect individuals who experience high psychological burden by the COVID-19 situation.

Mindfulness fosters self-efficacy and self-control that are important for the developing of functional problem-solving strategies in burdensome situations such as the COVID-19 outbreak. They could prevent dysfunctional strategies such as excessive SMU and compensate for a low PMH level [33,52]. Thus, both PMH and mindfulness could reduce the risk for addictive SMU. Persons with high PMH levels could be resilient enough to cope with the negative consequences of COVID-19 and not tend to excessive online activity. For them, a high level of mindfulness could result in an “oversaturation” with factors such as self-efficacy and therefore not further support the protective effect of PMH. In contrast, individuals with a low PMH level who experience the COVID-19 situation as especially burdensome could significantly benefit from mindfulness training that compensates their lack of self-efficacy, self-control, and functional coping strategies.

Against this background, public programs could focus on mindfulness training among the general population with a specific focus on individuals with low levels of PMH. Persons who are less motivated to engage in the training on their own could benefit from online group trainings via videotelephony (e.g., ZOOM, Skype). This could foster the feeling of social belonging and support despite social distancing. In particular young people, who are often prone to excessive SMU [53], could benefit from the programs. Moreover, individuals with enhanced depression and anxiety symptoms experience the COVID-19 outbreak as especially burdensome [54]. Often, they have low levels of PMH and tend to excessive media use to escape negative emotions [55]. However, this can foster their symptoms in the longer-term. Furthermore, for individuals who are in clinical treatment, a negative influence on the therapeutic process by addictive SMU was reported [29]. Therefore, an involvement of mindfulness training in the clinical context in addition to a screening for SMU intensity and its controlled reduction could be a promising strategy for an increase of treatment success during the pandemic and beyond.

The current study has some limitations. First, the relatively young, well-educated, and mostly female sample limits the generalizability of our findings to the general population. To assess their representativeness, the findings should be replicated in more gender-, age- and education-balanced groups. Second, due to the cross-sectional study design, only hypothetical conclusions on causality could be drawn from the present data. For true causal conclusions, present findings should be replicated by future experimental research with several measurement time points over a longer period of time. For example, it could be investigated whether a two-week mindfulness training can reduce addictive SMU and whether persons with low PMH levels especially benefit from this intervention immediately after it, one, three and six months later. Moreover, the focus of the present study was on whether mindfulness can moderate the relationship between PMH and addictive SMU. Our findings confirm this assumption. However, due to the cross-sectional study design, we cannot exclude other constellations of the investigated variables. For example, mindfulness could also moderate the link between COVID-19 burden and PMH, as well as the relationship between COVID-19 burden and addictive SMU. These constellations should also be investigated by future experimental studies. Third, we used only self-report measurements that are prone to same-source bias and social desirability [56,57]. Therefore, our results should be replicated by the assessment of and control for social desirability in the statistical analyses (e.g., Balanced Inventory of Social Desirability [56]). In addition, future studies should focus on additional data sources, for example, objective measures of psychological distress such as blood pressure. Yet, the assessment of such data could be impeded by the requirement for social distancing.

In conclusion, the present study reveals that the psychological burden caused by the COVID-19 outbreak could contribute to the development of addictive SMU. PMH and mindfulness could reduce this risk. Public programs should focus on the improvement of both protective factors. Thereby, especially individuals who experienced a decrease of PMH due to the COVID-19 burden could benefit from mindfulness trainings.

## Supporting information

S1 Dataset Dataset used for analyses in present study. (SAV)
